# Gender differences in measles incidence rates in a multi-year, pooled analysis, based on national data from seven high income countries

**DOI:** 10.1186/s12879-022-07340-3

**Published:** 2022-04-11

**Authors:** Manfred S. Green, Naama Schwartz, Victoria Peer

**Affiliations:** School of Public Health, University of Haifa, Abba Khoushy 199, Mount Carmel, 3498838 Haifa, Israel

**Keywords:** Measles, Sex differences, Incidence rate ratios, Meta-analysis

## Abstract

**Background:**

Gender differences in a number of infectious diseases have been reported. The evidence for gender differences in clinical measles incidence rates has been variable and poorly documented over age groups, countries and time periods.

**Methods:**

We obtained data on cases of measles by sex and age group over a period of 11–27 years from seven countries. Male to female incidence rate ratios (IRR) were computed for each year, by country and age group. For each age group, we used meta-analytic methods to combine the IRRs. Meta-regression was conducted to the estimate the effects of age, country, and time period on the IRR.

**Results:**

In the age groups < 1, 1–4, 5–9, 10–14, 15–44, and 45–64 the pooled IRRs (with 95% CI) were 1.07 (1.02–1.11), 1.10 (1.07–1.14), 1.03 (1.00–1.05), 1.05 (0.99–1.11), 1.08 (0.95–1.23), and 0.82 (0.74–0.92) respectively. The excess incidence rates (IR) from measles in males up to age 45 are remarkably consistent across countries and time-periods. In the age group 45–64, there is an excess incidence in women.

**Conclusions:**

The consistency of the excess incidence rates in young males suggest that the sex differences are more likely due to physiological and biological differences and not behavioral factors. At older ages, differential exposure can play a part. These findings can provide further keys to the understanding of mechanisms of infection and tailoring vaccination schedules.

## Introduction

Prior to immunization, measles was one of the commonest infectious diseases, affecting most children at an early age [1]. Despite the availability of an effective vaccine, achieving the goal of eradication remains elusive. Large epidemics continue to occur [2] and measles remains a major cause of childhood mortality, accounting for thousands of deaths annually [3]. The response to infection with measles is complex and not completely understood [4]. The virus replicates freely prior to the appearance of symptoms and clearance of viral RNA from blood and tissues takes some time after the rash disappears accompanied by a period of immunosuppression [4]. Following resolution of the infection, neutralizing antibodies persist and there is usually life-long immunity [4]. Based on serosurveys, the subclinical incidence of measles in very young children appears slightly higher than the incidence of clinically overt disease [5, 6]. A better understanding of the factors associated with developing clinical disease could contribute to determining optimal vaccine doses and schedules [7–9].

The reports on gender differences in clinical measles incidence rates have produced variable findings and there is poor documentation of such differences over age groups, countries and time periods [10–14]. The role of gender-related factors in measles morbidity could contribute to a better understanding of the mechanism of infection.

The main objective of the study was to use meta-analytic procedures to combine data from various countries at different times for different age groups, to obtain more stable estimates of the gender differences in measles incidence rates.

## Methods

### Source of data

National surveillance data on reported cases of measles, by age, sex and year, were obtained from relevant government institutions for seven countries from four continents: Europe (England, Germany, and Spain), Australasia (Australia and New Zealand), North America (Canada) and Asia (Israel). For Australia, for years 2001–2016, from the National Notifiable Diseases Surveillance System (NNDSS) [15], for Canada for the years 1991–2015, from the Public Health Agency of Canada (PHAC) [16], for England, for the years 1990–2016 directly from Public Health England (PHE), for Germany for the years 2001–2016, from the German Federal Health Monitoring System [17], for Israel from the Department of Epidemiology in the Ministry of Health for years 1991–2016, for New Zealand, for years 1997–2015 from the Institute of Environmental Science and Research (ESR) [18], and for Spain from the Spanish Epidemiological Surveillance for years 2005–2015 [19]. Information about the population size by age, sex and year was obtained for Australia from ABS.Stat [20] (Australia’s Bureau of statistics), for Canada from Statistics [21], Canada, CANSIM database, for England, from the Population Estimates Unit, Population Statistics Division, Office for National Statistics [22], for Germany from the German Federal Health Monitoring System [23], for Israel from the Central Bureau of Statistics [24], for New Zealand from Statistics New Zealand [25] and for Spain from the Department of Economic and Social Affairs, Population Division [26].

### Statistical analyses

Measles incidence rates (IR) per 100,000 were calculated by sex, age group, for each country and calendar year using the number of reported cases divided by the respective population size and multiplied by 100,000. The age groups considered were < 1 year (infants), 1–4 (early childhood), 5–9 (late childhood), 10–14 (puberty), 15–39/44 (young adulthood) and 40–59 or 45–64 (middle adulthood). Surveillance system in Canada and New Zealand, used similar age-groups except for the following: 15–39 and 40–59. For Australia data for infants and 1–4 aged children disaggregated by sex are missing. The male to female incidence rate ratio (IRR) was calculated by dividing the incidence rate in males by that of females, by age group, country and time period.

### Meta-analytic methods

As in previous studies of gender differences in infectious diseases [27–30], we used meta-analytic methods to establish the magnitude and consistency of the gender differences in the incidence of measles (disaggregated by age group, across different countries and a number of years). Meta-analysis is a statistical technique for summarizing the data from several sources into a weighted average. This method was used to generate single, quantitative estimates of the gender differences in the incidence of measles for each age group, combining data from a number of countries and time periods. The method provides improved power for determining more stable estimates of the outcome measure. The outcome measure in this study was the male to female IRR. For each age group, the IRRs for each country were pooled and then the pooled IRRs for each country were combined. Forest plots with the pooled IRRs, for the countries and years of reporting, were prepared separately for the six age groups. Heterogeneity of the IRRs was evaluated using the Q statistic and I^2^ was calculated as an estimate of the percentage of between-study variance. If the p-value for the Q statistic was < 0.05, or I^2^ was higher than 50%, the random effects models was used. Otherwise, the fixed effects model was considered, although due to the low power of the Q statistic, the more conservative random effects model was usually preferred. In order to explore the contribution of other variables as countries and the reported years, meta-regression analyses were performed. Leave-one-out sensitivity analysis was performed to estimate the effect of variables as different countries and years on the male to female incidence risk ratio. The meta-analyses and meta-regressions were carried out using STATA software version 12.1 (Stata Corp., College Station, TX).

## Results

### Descriptive statistics

The data on the measles incidence rates per 100,000 populations, by countries, sex and age groups are presented in Table 1. There were significant differences in the absolute incidence rates in in England and New Zealand, where the rates were high.

**Table 1:** Details of the countries included in the meta-analysis, by sex and age group—descriptive data

Age	Country	Years	Male n/N	Male IR	Female n/N	Female IR	IRR
< 1	Canada	1991–2015	359/4682619	7.67	335/4446799	7.53	1.02
	England	1990–2016	15,249/8725051	174.77	14,034/8306732	168.95	1.03
	Germany	2001–2016	545/5740478	9.49	507/5448550	9.31	1.02
	Israel	1991–2016	190/1770100	10.73	129/1680000	7.68	1.40
	New Zealand	1997–2015	413/576900	71.59	319/548520	58.16	1.23
	Spain	2005–2015	426/2679186	15.90	378/2514548	15.03	1.06
1–4	Canada	1991–2015	902/19156418	4.71	843/18225737	4.63	1.02
	England	1990–2016	29,345/34821935	84.27	25,789/33207057	77.66	1.09
	Germany	2001–2016	2908/23509315	12.37	2569/22311030	11.51	1.07
	Israel	1991–2016	1026/6843500	14.99	762/6500900	11.72	1.28
	New Zealand	1997–2015	636/2308880	27.55	532/2191980	24.27	1.13
	Spain	2005–2015	717/10880587	6.59	620/10233932	6.06	1.09
5–9	Australia	2001–2016	60/11398585	0.53	62/10814642	0.57	0.92
	Canada	1991–2015	1956/24668602	7.93	1878/23469919	8.00	0.99
	England	1990–2016	11,013/42989082	25.62	10,039/41012194	24.48	1.05
	Germany	2001–2016	2814/30760941	9.15	2737/29187252	9.38	0.98
	Israel	1991–2016	414/7977400	5.19	343/7580100	4.53	1.15
	New Zealand	1997–2015	284/2899540	9.79	270/2752910	9.81	1.00
	Spain	2005–2015	201/13017097	1.54	224/12287011	1.82	0.85
10–14	Australia	2001–2016	97/11377822	0.85	81/10797396	0.75	1.14
	Canada	1991–2015	2143/25685783	8.34	2076/24391864	8.51	0.98
	England	1990–2016	6215/42597565	14.59	5169/40624659	12.72	1.15
	Germany	2001–2016	2134/33455166	6.38	2106/31724889	6.64	0.96
	Israel	1991–2016	273/7398300	3.69	251/7029400	3.57	1.03
	New Zealand	1997–2015	216/2919850	7.40	155/2776650	5.58	1.33
	Spain	2005–2015	231/12301238	1.88	241/11627137	2.07	0.91
15–39/44	Australia	2001–2016	590/73591102	0.80	496/72741755	0.68	1.18
	Canada	1991–2015	2366/143987472	1.64	2061/140453550	1.47	1.12
	England	1990–2016	6905/234901126	2.94	5079/233399206	2.18	1.35
	Germany	2001–2016	3801/257895408	1.47	3663/247590330	1.48	1.00
	Israel	1991–2016	746/35538900	2.10	654/35142900	1.86	1.13
	New Zealand	1997–2015	472/13546700	3.48	459/13976900	3.28	1.06
	Spain	2005–2015	1697/110542308	1.54	1551/105413400	1.47	1.04
45–64	Australia	2001–2016	18/41988401	0.04	20/42573071	0.05	0.91
(40–59)	Canada	1991–2015	74/110461323	0.07	93/109655649	0.08	0.79
	England	1990–2016	1284/175100277	0.73	1586/177644620	0.89	0.82
	Germany	2001–2016	179/169811651	0.11	223/169907321	0.13	0.80
	Israel	1991–2016	64/14322400	0.45	62/15453800	0.40	1.11
	New Zealand	1997–2015	32/10201030	0.31	26/10685350	0.24	1.29
	Spain	2005–2015	31/63103755	0.05	33/64340310	0.05	0.96

Infants =  < 1 year; early childhood = 1–4 years; late childhood = 5–9 years; puberty = 10–14 years; young adulthood = 15–44 or 15–39 years; middle adulthood = 40–59 or 45–64 years;

*IR* incidence rate, IR per 100 000 Male or Female population; *IRR* male: female Incidence Rate Ratio; *n* cumulative number of pertussis cases for given years; *N* cumulative number of the population for given years

### Meta-analytic analyses

**Fig. 1:**
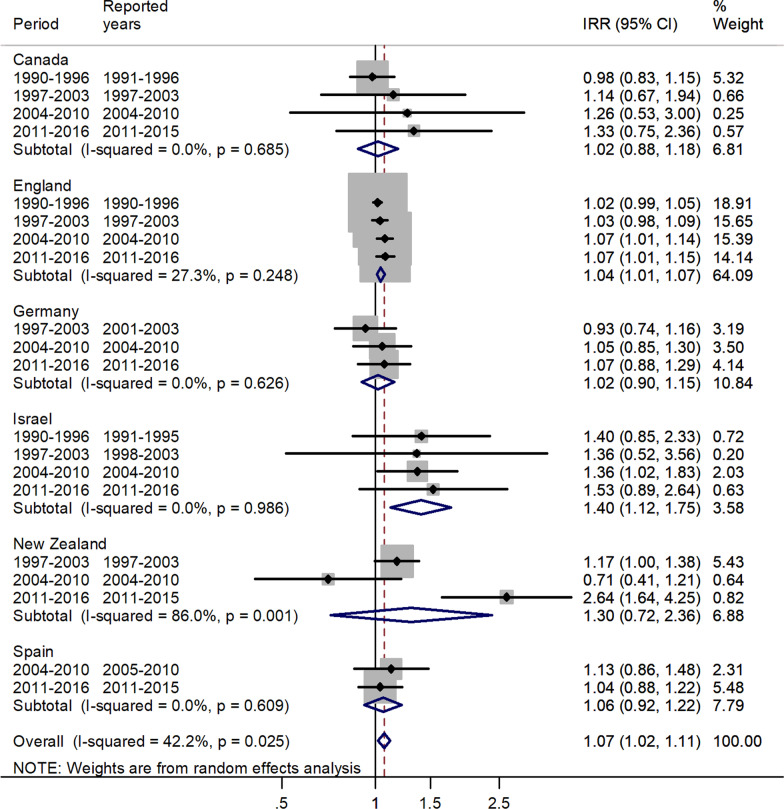
Forest plot of the male to female measles IRRs for different years in Canada, England, Germany, Israel, New Zealand, and Spain in infants (< 1 years)

**Fig. 2:**
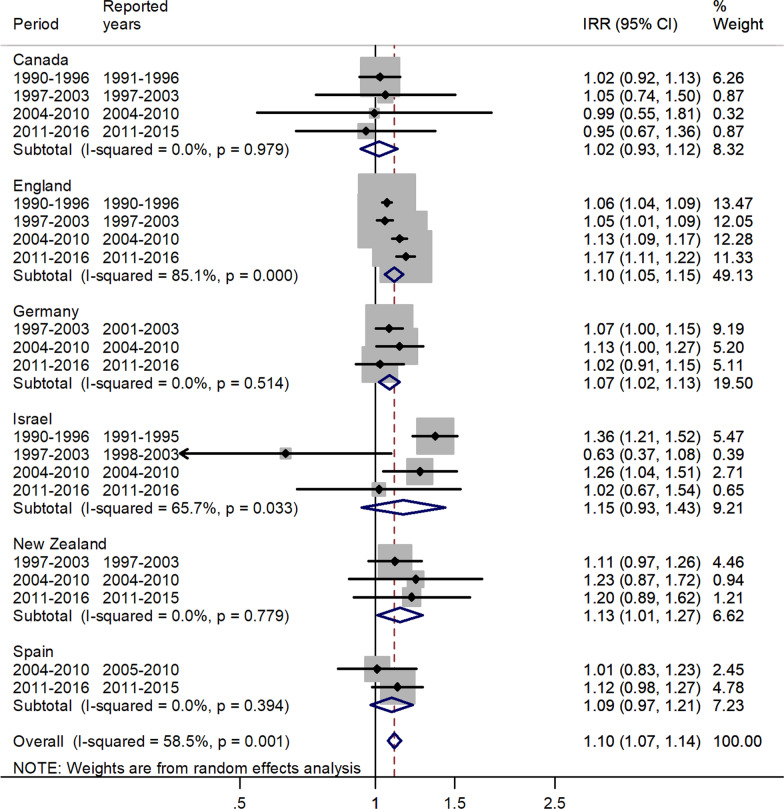
Forest plot of the male to female measles IRRs for different years in Canada, England, Germany, Israel, New Zealand, and Spain for age 1–4 years

**Fig. 3:**
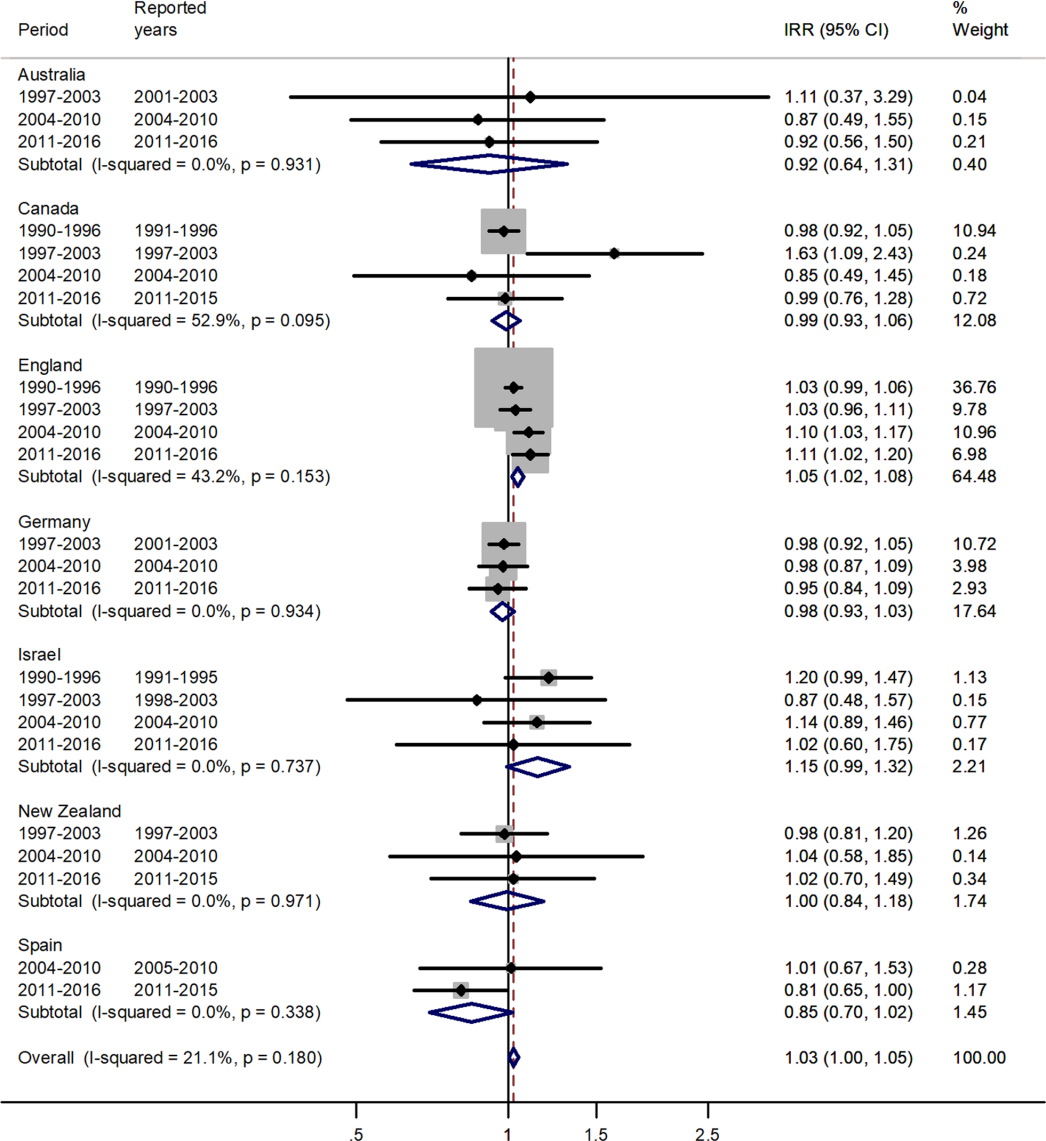
Forest plot of the male to female measles IRRs for different years in Australia, Canada, England, Germany, Israel, New Zealand, and Spain for age 5–9 years

**Fig. 4:**
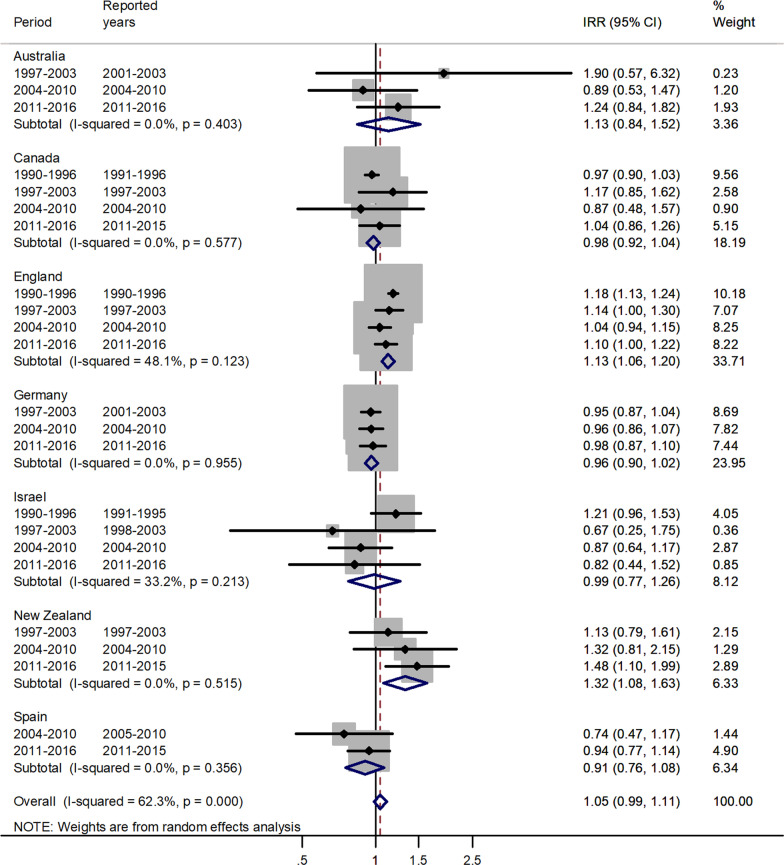
Forest plot of the male to female measles IRRs for different years in Australia, Canada, England, Germany, Israel, New Zealand, and Spain for age 10–14 years

**Fig. 5:**
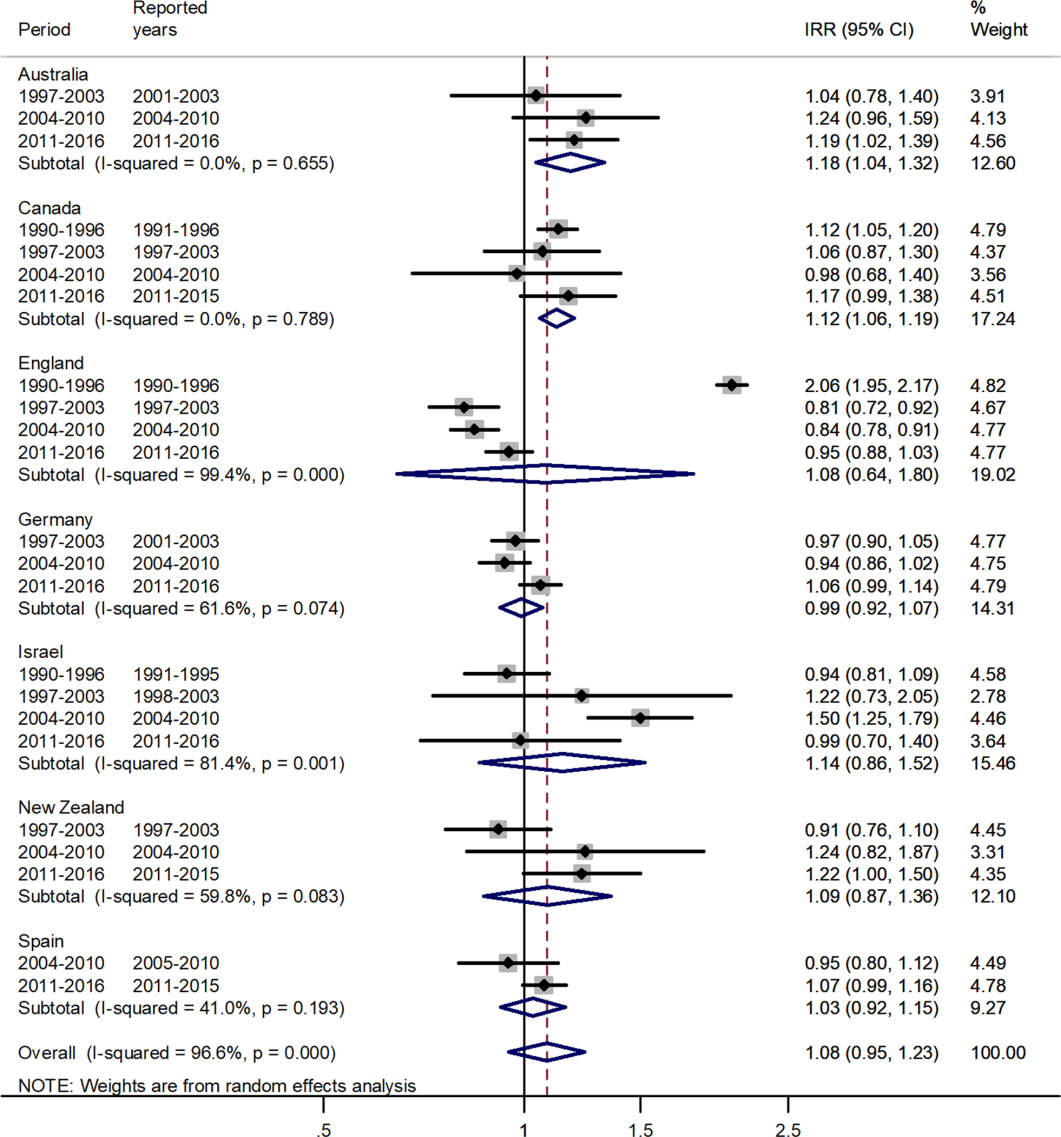
Forest plot of the male to female measles IRRs for different years in Australia, Canada, England, Germany, Israel, New Zealand, and Spain for age 15–44 (or 15–39) years

**Fig. 6:**
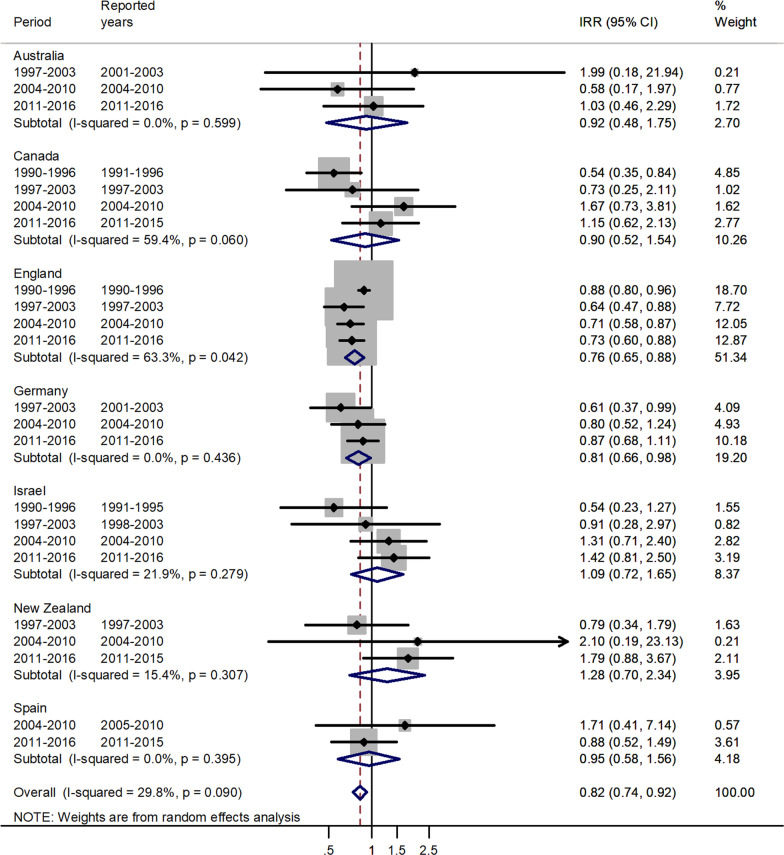
Forest plot of the male to female measles IRRs for different years in Australia, Canada, England, Germany, Israel, New Zealand, and Spain for age 45–64 (or 40–59) years

The forest plots for the pooled estimates using meta-analytic methods are shown by age group in Figs. 1, 2, 3, 4, 5, 6. The forest plot for infants is shown in Fig. 1.

There was an excess in incidence rates among males, with a pooled male to female IRR = 1.07, 95% CI 1.02–1.11, which varied between 1.02 in Canada and Germany to 1.40 in Israel.

The forest plot for the age 1–4 is shown in Fig. 2.

There was an excess in incidence rates for males with a pooled IRR = 1.10 and 95% CI 1.07–1.14, and ranged between 1.02 in Canada and 1.15 in Israel.

The forest plot for age 5–9 is given in Fig. 3.

There was an excess in incidence rates in males compared with females, with a pooled IRR = 1.03 and 95% CI 1.00–1.05. Subtotal IRR's varied between 0.85 in Spain to 1.15 in Israel.

The forest plot for age 10–14 is given in Fig. 4.

In the age group 10–14, there was an excess in incidence rates among males, with a pooled IRR = 1.05 and 95% CI 0.99–1.11. The IRR’s varied from 0.91 in Spain to 1.32 in New Zealand.

The forest plot for age 15–44 is given in Fig. 5.

For age 15–44, there was an excess in incidence rates in males with a pooled IRR = 1.08 and 95% CI 0.95–1.23. The IRR’s varied from 0.99 in Germany to 1.18 in Australia.

The forest plot for age 45–64 is given in Fig. 6.

For age 45–64 or 40–59, there was an excess in incidence rates in females with a pooled male to female IRR = 0.82 and 95% CI 0.74–0.92. The IRR’s varied from 0.76 in England to 1.28 in New Zealand. The numbers in the age group 65+ were too small for meaningful estimates.

A meta-regression analysis showed that most of the variance in the IRRs was contributed by the age groups, with some differences between countries and time periods. To evaluate the effect of individual countries on the male to female incidence ratios, we performed leave-one-out sensitivity analysis and recomputed the pooled IRRs (presented in Tables 2 and 3).

**Table 2:** Sensitivity analysis for countries, by age group

Age group	0–1 IRR (CI)	1–4 IRR (CI)	5–9 IRR (CI)	10–14 IRR (CI)	15–44 IRR (CI)	45–64 IRR (CI)
Country removed						
Australia	–	–	1.03 (1.004–1.05)	1.04 (0.95–1.15)	1.11 (0.98–1.26)	0.85 (0.77–0.94)
Canada	1.1 (1.01–1.2)	1.12 (1.06–1.17)	1.03 (1.01–1.05)	1.07 (0.95–1.19)	1.12 (0.98–1.28)	0.86 (0.78–0.96)
England	1.11 (1.005–1.24)	1.11 (1.03–1.2)	0.99 (0.95–1.02)	1.01 (0.94–1.08)	1.08 (1.02–1.14)	0.89 (0.77–1.03)
Germany	1.11 (1.01–1.22)	1.11 (1.04–1.19)	1.04 (1.01–1.06)	1.07 (0.97–1.18)	1.15 (1.03–1.28)	0.87 (0.77–0.99)
Israel	1.05 (1.0002–1.1)	1.08 (1.07–1.1)	1.02 (1.0002–1.05)	1.05 (0.95–1.16)	1.12 (0.99–1.27)	0.83 (0.77–0.88)
New Zealand	1.06 (0.99–1.13)	1.1 (1.04–1.16)	1.03 (1.003–1.05)	1.02 (0.93–1.12)	1.13 (1.001–1.28)	0.83 (0.78–0.89)
Spain	1.1 (1.002–1.2)	1.11 (1.05–1.17)	1.03 (1.01–1.05)	1.07 (0.97–1.18)	1.13 (1.0004–1.29)	0.85 (0.77–0.93)

*IRR* incidence rate ratio; *CI* confidence interval

**Table 3:** Sensitivity analysis for time periods, by age group

Age group	0–1 IRR (CI)	1–4 IRR (CI)	5–9 IRR (CI)	10–14 IRR (CI)	15–44 IRR (CI)	45–64 IRR (CI)
Years removed						
1990–1996	1.07 (1.03–1.1)	1.11 (1.05–1.16)	1.03 (1.0004–1.06)	1.02 (0.98–1.06)	0.98 (0.91–1.06)	0.78 (0.69–0.9)
1997–2003	1.05 (1.005–1.11)	1.11 (1.06–1.16)	1.03 (1.004–1.05)	1.05 (0.99–1.13)	1.16 (0.85–1.59)	0.85 (0.79–0.9)
2004–2010	1.04 (1.001–1.09)	1.08 (1.04–1.13)	1.02 (0.99–1.04)	1.07 (1.02–1.12)	1.16 (0.85–1.59)	0.82 (0.72–0.92)
2011–2016	1.04 (1.003–1.07)	1.08 (1.04–1.12)	1.02 (1.001–1.05)	1.04 (0.97–1.12)	1.12 (0.77–1.62)	0.79 (0.69–0.91)

*IRR* incidence rate ratio; *CI* confidence interval

After omitting each country (one country at a time), the pooled IRR’s were very similar. After omitting a group of years at a time, the pooled incidence RR’s still remained largely unchanged. Thus, no single country or group of years substantially affected the pooled IRRs. This confirms that the results are stable and robust. The changes in effect size are likely due to the relatively small numbers in the sub-groups. Sensitivity analysis by age group and country and by clusters of years were performed. No single country, including England or group of years affected the pooled IRRs.

## Discussion

Based on meta-analytic analyses of national data from seven countries, over a period of 11–27 years, we found that the incidence rates of clinical measles were 7%, 10%, 3% and 5% significantly higher in males in infancy, ages 1–4, 5–9 and 10–14, respectively. In adults, the picture was less clear. In the age group 15–44, it was 8% higher in males, but with a wide confidence interval and not statistically significant. At age 44–64, the incidence rates were 18% lower in males. While the sex differences observed are not great, it has been shown that in infectious diseases where the clinical to subclinical ratio is relatively high, the observed gender differences tend to be lower in magnitude [31]. The variation in the absolute incidence rates can be explained, at least in part, by the occurrence of local outbreaks. For example during 1 year period, from 2012 to 2013, there were a series of outbreaks in England [10, 11]. New Zealand has suffered outbreaks due to measles importation [12].

Although a male predominance of measles incidence rates has been reported [32], to the best of our knowledge, there are no reports that combine data from different countries and time periods for different age groups. This study has several strengths. The study is based on national, notifiable diseases surveillance data with large total populations and numbers of cases. There should be minimal selection bias since we used national data for long periods for each country. A possible limitation is that there are likely to be differences between countries in diagnosis and reporting of measles. However, we do not believe that diagnosis or reporting differs between males and females, since the countries in this study do not have a culture of giving differential treatment according to sex. However, there could be differences between sexes in the use of health services. Since there is evidence that females tend to use health services more than males [33], the excess rates observed in males may underestimate of the true excess in the incidence rates in males. Differences in vaccination rates and national vaccination policies between countries should not affect sex-related differences in measles incidence. This pooled data analysis from seven countries does not include countries from Africa and Asia and thus, the findings may be generalizable only to high-income countries.

In this study we pooled age disaggregated data from seven countries, over a number of time-periods. As in previous studies of infectious diseases such as viral meningitis, shigellosis and campylobacteriosis, we used meta-analytic methods to pool the male to female IRRs over countries and time periods for separate age groups [27, 28, 30]. As in the current study, we found an excess in incidence rates in males at young ages [27, 28]. Pertussis is a notable exception, where females have been found to consistently have higher incidence rates at all ages [29].

It is of interest to note that sex differences in measles mortality rates have been reported. In one multi-national study, mortality from measles was examined in the WHO reports on causes of death in different countries over many years since 1950 [34]. The pooled results showed excess female mortality under the age of 50. However, in other studies, case-fatality rates were slightly higher for males of at ages [35, 36]. An interesting phenomenon is the excess mortality in females following the use of high-titer measles containing vaccines [37, 38]. Atabani et al. [39] suggested that this may be related to sex differences in antibody dependent cellular cytotoxicity, which they found to be lower in females following measles vaccination.

The exact mechanisms underlying the excess measles incidence rates in young males found in the current study are not clear and are likely to be multi-factorial. This study cannot address the mechanisms. However, it is possible to speculate on some possibilities. These include exposure differences, response to vaccines, genetic and hormonal factors. In infants and early childhood, it is unlikely that the sex differences in incidence rates are due to differences in exposure. At older ages, since females are generally over-represented as caregivers for sick children in healthcare or day-care facilites, one might expect higher incidence rates in the older age groups. This was not observed in the current study, except in the age group 45–64. Prior to the introduction of immunization in the late 1950’s, almost all children acquired measles, so measles in adults was uncommon. Measles in adults who have been immunized may be due to waning of immunity over time. While the measles vaccine is highly effective in preventing disease, gender differences have been found in response to measles vaccines. A study of children and adults in Spain found that females develop significantly higher measles IgG titers following vaccination in compare to age-matched males [40]. There is evidence of a weaker antibody response to measles vaccine in boys [41] and a higher humoral antibody response to live measles vaccine in females has been observed in young adults [42]. There also appears to be less waning in immunity to measles vaccine in females [43]. Thus, even after immunization, males may be more susceptible to measles than females.

Sex differences in measles incidence rates may be related to the imbalance in the expression of genes encoded on the X and Y-chromosomes of a host. The phenomenon of X chromosome inheritance and expression is a cause of immune disadvantage of males and the enhanced survival of females following immunological challenges [44]. The possible role of sex hormones remains largely unknown. Changes in male-to-female morbidity ratios with age may reflect differences between the sexes in immune and endocrine systems [45].

Estrogen may modulate cytokine production in vivo and contribute to sex-related differences in immune responses [46, 47]. Both, CD8+ and CD4+ T cells, which play significant roles during the rash phase of measles, express estrogen receptors (ERa and ERb) and are estrogen sensitive [48]. The surge in sex hormone levels in infancy that mimics sex steroid levels at puberty (‘minipuberty’) could affect immune cells differently in boys and girls and influence maturation of the immune system [49]. This transient rise in sex steroid levels may also impact on immune cells differently between boys and girls at later ages [50] and could play a role in the observed excess in incidence rates in males.

## Conclusions

This study provides stable estimates of the magnitude of the excess male incidence rates in measles in most age groups, particularly in infants and children. Despite the generally high clinical to sub-clinical ratio, which may mask subtle sex differences in the incidence of clinical disease, the excess in young males is remarkably stable over a number of countries and for a period of around 20 years. A better understanding of the gender differences in disease incidence can help to elucidate genetic and hormonal determinants of measles infection and should be taken into account in the tailoring of vaccine doses and schedules.

## Data Availability

All data are available from the original sources or from the authors. The datasets analyzed during the current study are available from the corresponding author on reasonable request. For all countries, except Israel and England, public access to the databases is open and links are part of the references list. We received administrative permission from the official representative of Israeli Ministry of Health and Public Health England (PHE) to use the data for publication.
